# Magmatic evolution of the Kikai caldera revealed by zircon triple dating and its chemistry

**DOI:** 10.1038/s41598-025-87264-5

**Published:** 2025-01-23

**Authors:** Hisatoshi Ito

**Affiliations:** Central Research Institute of Electric Power Industry, Chiba, 270-1194 Japan

**Keywords:** Geochemistry, Volcanology, Natural hazards

## Abstract

Reconstructing the volcanic history of the Kikai caldera, a large active volcano that produced a ~ 160 km^3^ eruption at 7.3 ka off the southern coast of Kyushu Island (southwest Japan), is crucial to assess potential future volcanic hazards at both regional and global scales. However, revealing its volcanic history before the 7.3 ka eruption has been challenging due to the caldera being mostly submerged. In this study, we present evidence that the Kikai caldera erupted a geochemically distinct silicic lava at ~ 250 ka by using zircon triple (U-Pb, Th-Pb, U-Th) dating and its chemistry. The presence of 1.5–1.0 Ma zircons in the 7.3 ka eruption deposits suggests that zircon crystallization in the Kikai caldera began during this period. We further infer large eruptions occurred around 0.7–0.6 Ma, suggesting that the Kikai caldera may have experienced at least 5 major eruptions during its 1.0–1.5-million-year magmatic evolution.

**Supplementary Information:**

The online version contains supplementary material available at 10.1038/s41598-025-87264-5.

## Introduction

Elucidating magmatic evolution of Quaternary caldera-forming volcanoes is crucial for humankind to assess whether another gigantic eruption may happen in near future. Numerous such efforts have been performed on various volcanoes such as Yellowstone caldera^1^, Long Valley caldera^2^, both in the USA, Toba caldera^3–5^, Indonesia, and Taupo Volcanic Zone^6^, New Zealand, to name a few.

**Fig. 1:**
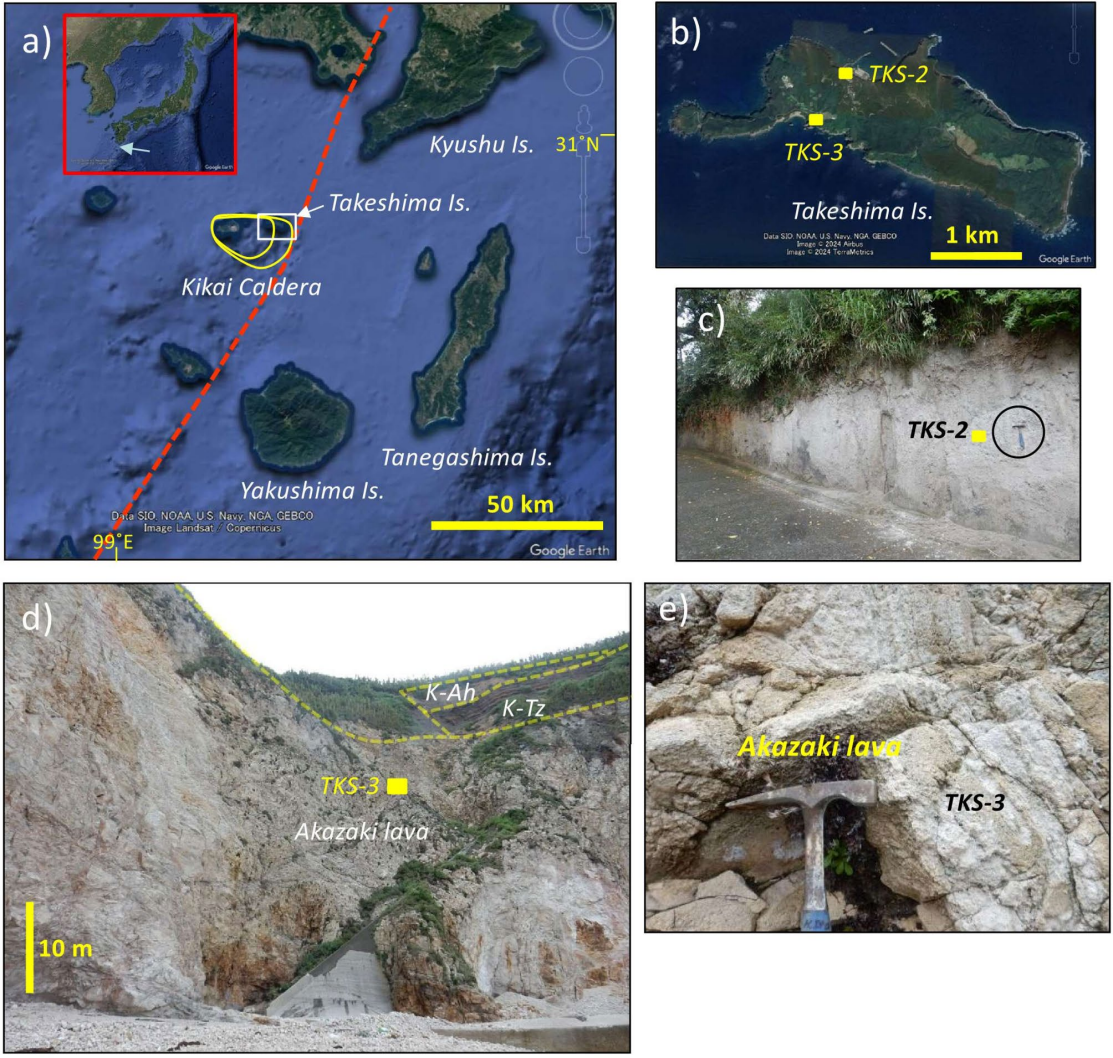
Location of the Kikai caldera. (**a**) Inner and outer Kikai caldera rims are shown by solid yellow lines. A red dashed line shows a volcanic front, which is the frontal border of a volcanic belt in an arc setting. An arrow in inset shows the location of the Kikai caldera in Japan. The location of map (**b**) is shown in an open white rectangle. (**b**) Takeshima Island with sampling locations of K-Ah (TKS-2) and Akazaki lava (TKS-3). (**c**) An outcrop of the Takeshima pyroclastic flow (K-Ah) where TKS-2 was sampled. A hammer in a circle is ~ 30 cm. (**d**) A cliff mostly composed of the Akazaki lava where TKS-3 was sampled. Lithological boundaries are shown by dashed yellow lines. K-Tz: Nagase pyroclastic flow from the 95 ka K-Tz eruption. K-Ah: Takeshima pyroclastic flow from the 7.3 ka K-Ah eruption. (**e**) Close-up view of the sample TKS-3. Sampling locations are shown in yellow rectangles and samples were obtained near the hammer in (**c**) and (**e**). (**a**) and (**b**) adapted from Google Earth Pro, version 7.3.6.9796 (https://www.google.com/earth/about/versions/). Map data: (**a**) Image Landsat/Copernicus, (**b**) Image ©2024 Airbus.

 The Kikai caldera, off the southern coast of Kyushu Island, southwest Japan (Fig. 1), has experienced at least three caldera-forming eruptions in the Quaternary (Table 1): the ~ 140 ka Koabi (K-ab), the 95 ka Kikai-Tozurahara (K-Tz) and the 7.3 ka Kikai-Akahoya (K-Ah) eruptions^7–9^. The latest 7.3 ka K-Ah eruption categorized once as a supereruption^10^ was one of the largest eruptions on Earth during the Holocene, which produced a dense rock equivalent (DRE) volume of ~ 160 (133–183) km^3^^11^, leading to the formation of the present Kikai caldera (17-km wide and 20-km long)^8^. The latest two large eruptions (K-Tz, K-Ah) caused widespread ash dispersal across the Japan archipelago, which have come to be known as important marker tephras^12^.

**Table 1:** Eruption history in the Kikai caldera.

Tephra & lava name	Abbreviation	Magma type	DRE volume (km^3^)	Age (ka)	Reference
Akahoya	K-Ah	Caldera-forming (silicic)	133–183	7.3	Shimizu et al.^11^
Komorikô tephra group	K-Km	Basaltic to andesitic (?)		13–8	Ono et al.^14^; Okuno^41^
Tozurahara	K-Tz	Caldera-forming (silicic)	130	95	Machida et al.^7^
Koabi	K-ab	Caldera-forming (silicic)	~130	~140	Machida et al.^7^
Akazaki lava		Silicic		~250	This study
Koseda	Ksd	Caldera-forming (silicic)		~630	Ito et al.^15^
Anbo	Anbo	Caldera-forming (silicic)		~730	Ito et al.^15^

Although the volcanic history after the 95 ka K-Tz eruption of the Kikai caldera is fairly well-investigated^8,9,13^, those before this time are not. The ~ 140 ka K-ab eruption products were only documented at subaerial parts of the Kikai caldera (Satsuma-Iwo-jima and Takeshima islands) as thick (> 100 m) mostly welded pyroclastic flow deposits^14^. Before the ~ 140 ka K-ab eruption, there have been no caldera-forming eruption products documented in the Kikai caldera itself. Two widespread tephras (Koseda (Ksd) and Anbo), dated at ~ 630 ka and ~ 730 ka by zircon U-Pb^15^, were assumed to be derived from the Kikai caldera, only because zircons coeval with these tephras were found as xenocrysts in the 95 ka K-Tz tephra in Yakushima Island, situated ~ 30 km south of the Kikai caldera (Fig. 1). Apart from these eruptions related to caldera formation, a thick (> 60 m) silicic lava flow, Akazaki lava, is documented^14^ on the Takeshima Island (Fig. 1). The Akazaki lava, stratigraphically below the K-Tz (Fig. 1d), was once thought to have been deposited in the pre-caldera stage, i.e., older than ~ 700 ka^14,16^, while no radiometric ages were reported, supposedly due to intense silicification reducing the chance of finding minerals suitable for K-Ar or Ar-Ar dating. Here, two samples (K-Ah, Akazaki lava) were obtained in the Takeshima Island (Fig. 1) for zircon geochronology and geochemistry to further reveal the volcanic history of the Kikai caldera.

## Results

### U-Pb age

Twenty-five zircon grains from the K-Ah were dated during the first experiment in 2017. Of them, only two passed the common Pb criteria of ≤ 75% using the shallow section (~ 8–16 μm depth of zircon) while seven grains passed them using the deep section (~ 16–24 μm depth). Therefore, we report zircon U-Pb and Th-Pb ages using the deep section hereafter.

**Fig. 2:**
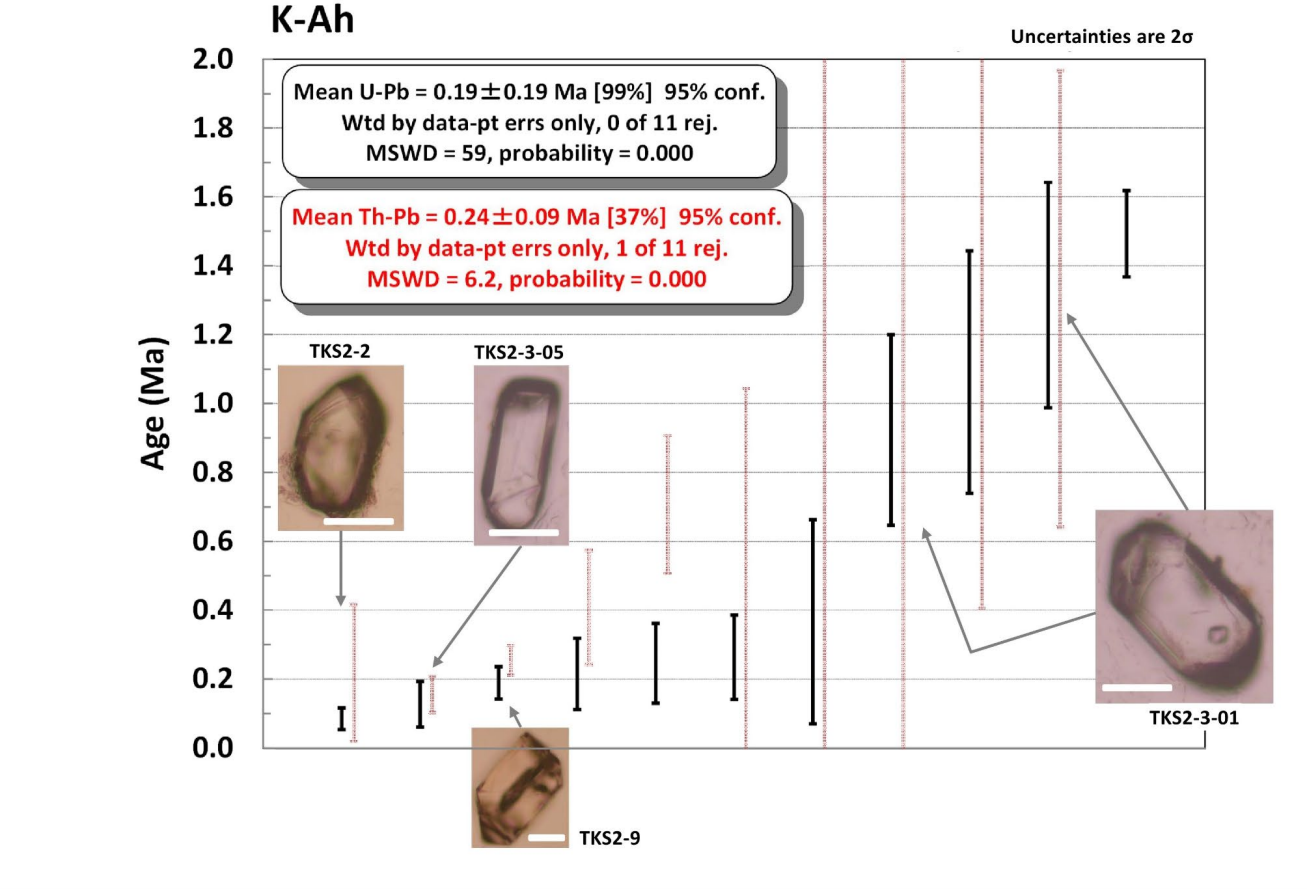
K-Ah zircon individual U-Pb (^238^U-^206^Pb) and Th-Pb (^232^Th-^208^Pb) ages arranged in rank order for U-Pb data. Th-Pb ages are in red and next to the corresponding U-Pb ages. Because most of Th-Pb ages have a large uncertainty, they are shown in pale red. Selected zircon images with arrows pointing to the corresponding age are shown. Corresponding cathodoluminescence images are shown in Fig. S3. Note zircon ‘TKS2-3-01’ was dated twice. White bar scale is 50 μm.

In total, 35 zircons from the K-Ah were dated and 11 grains passed the common Pb criteria over 3 experiments. Of the 11 grains, one grain was dated twice and both passed the common Pb criteria, and hence 12 data passed the criteria. The individual U-Pb age varied from 0.09 ± 0.03 Ma to 6.32 ± 2.08 Ma (Table S1). Here, individual grain age errors are shown as 2σ while weighted mean ages are shown as 95% or 97.9% confidence levels, which are nearly equivalent to 2σ and therefore we treat these as 2σ hereafter. Also, age errors are shown as 2σ hereafter in the text unless specified. Because the age of 6.32 ± 2.08 Ma was exceptionally older than the other 10 grain ages and the age was in poor quality (barely passed the common Pb criteria), we neglect this age hereafter and the remaining 11 age data were plotted (Fig. 2). The weighted mean age of 0.19 ± 0.19 Ma with a large mean square weighted deviation (MSWD) of 59 indicates that detrital (or xenocrystic) zircons were incorporated. Therefore, the weighted mean age is not valid. A grain (sample: TKS2-3-01) was dated twice, yielding an unpolished zircon age of 1.31 ± 0.33 Ma and a polished zircon age of 0.92 ± 0.28 Ma, which are consistent within uncertainty.

**Fig. 3:**
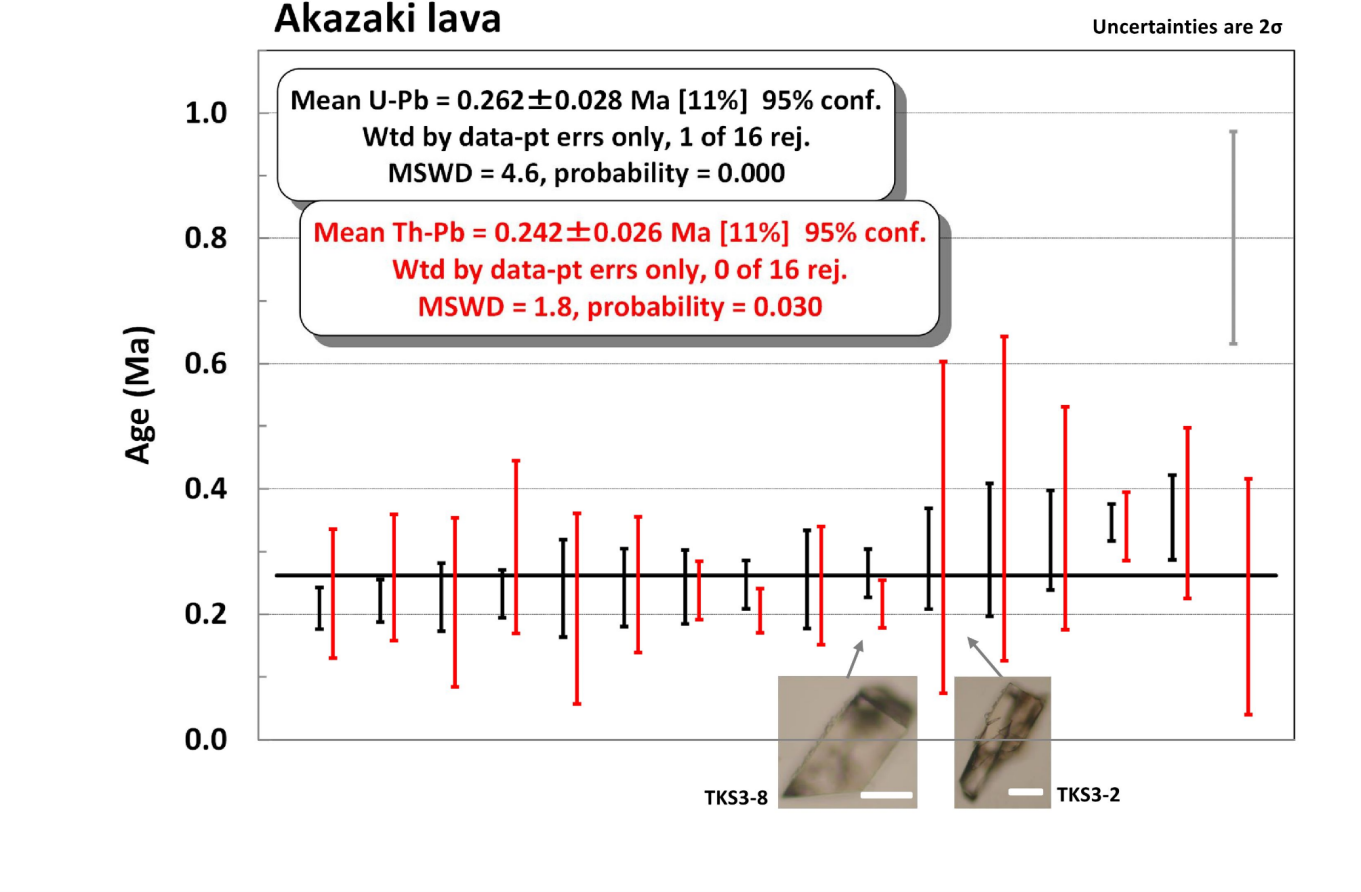
Akazaki lava zircon individual U-Pb (^238^U-^206^Pb) and Th-Pb (^232^Th-^208^Pb) ages arranged in rank order for U-Pb. Th-Pb ages are in red and arranged next to the corresponding U-Pb ages. A black horizontal bar shows the mean age of U-Pb. A gray vertical bar is omitted for the mean calculation. Selected zircon images with arrows pointing to the corresponding age are shown. Corresponding cathodoluminescence images are shown in Fig. S3. White bar scale is 50 μm.

Twenty-eight U-Pb grain ages were obtained from the Akazaki lava. Of them, 16 grains passed the common Pb criteria, yielding ages from 0.21 ± 0.03 Ma to 0.80 ± 0.17 Ma (Table S1). Excluding the oldest 0.80 ± 0.17 Ma as an outlier, a weighted mean age of 0.26 ± 0.03 Ma (*n* = 15, MSWD = 4.6) was obtained (Fig. 3).

All the three reference zircons of Plešovice^17^, Bishop Tuff^18^, and Fish Canyon Tuff^19^ yielded ages consistent with their reference ages (Table S1), corroborating the validity of the dating procedure. Fig. S1 shows individual zircon U-Pb ages for the Bishop Tuff, which yielded a weighted mean age of 0.77 ± 0.02 Ma (*n* = 9, MSWD = 1.6).

### Th-Pb age

Because the common Pb criteria for Th-Pb dating was adopted as the same with that for U-Pb dating (see “Methods”), 11 grains passed the criteria for the K-Ah zircon, yielding Th-Pb ages from 0.16 ± 0.05 Ma to 8.77 ± 10.77 Ma (Table S1). Figure 2 shows individual Th-Pb ages next to corresponding U-Pb ages. A weighted mean Th-Pb age of 0.24 ± 0.09 Ma with a MSWD of 6.2 was obtained.

Similarly, Fig. 3 shows individual Th-Pb ages next to corresponding U-Pb ages for the Akazaki lava zircon, yielding a weighted mean Th-Pb age of 0.24 ± 0.03 Ma (*n* = 16, MSWD = 1.8), which is in good agreement with its weighted mean U-Pb age of 0.26 ± 0.03 Ma.

As for the three reference zircons, both the Bishop Tuff and the Fish Canyon Tuff yielded ages consistent with their reference ages (Table S1), whereas the Plešovice yielded a considerably older weighted mean Th-Pb age of 384.0 ± 19.0 Ma (*n* = 16, MSWD = 45). Fig. S1 shows individual Th-Pb ages next to corresponding U-Pb ages for the Bishop Tuff zircon, yielding a weighted mean Th-Pb age of 0.78 ± 0.08 Ma (*n* = 8, MSWD = 5.0).

### U-Th age

Most of zircons (10 out of 11) from the K-Ah tephra yielded poor quality data (either < 30 counts per second (cps) of ^230^Th or > 50% (1σ) of model age uncertainty) (Table S2). The only one zircon (sample: TKS2-3–05) that passed the criteria yielded a U-Th age of 53 ± 26 ka (1σ) assuming Th/U of magma (Th/U_magma_) of 4.

**Fig. 4:**
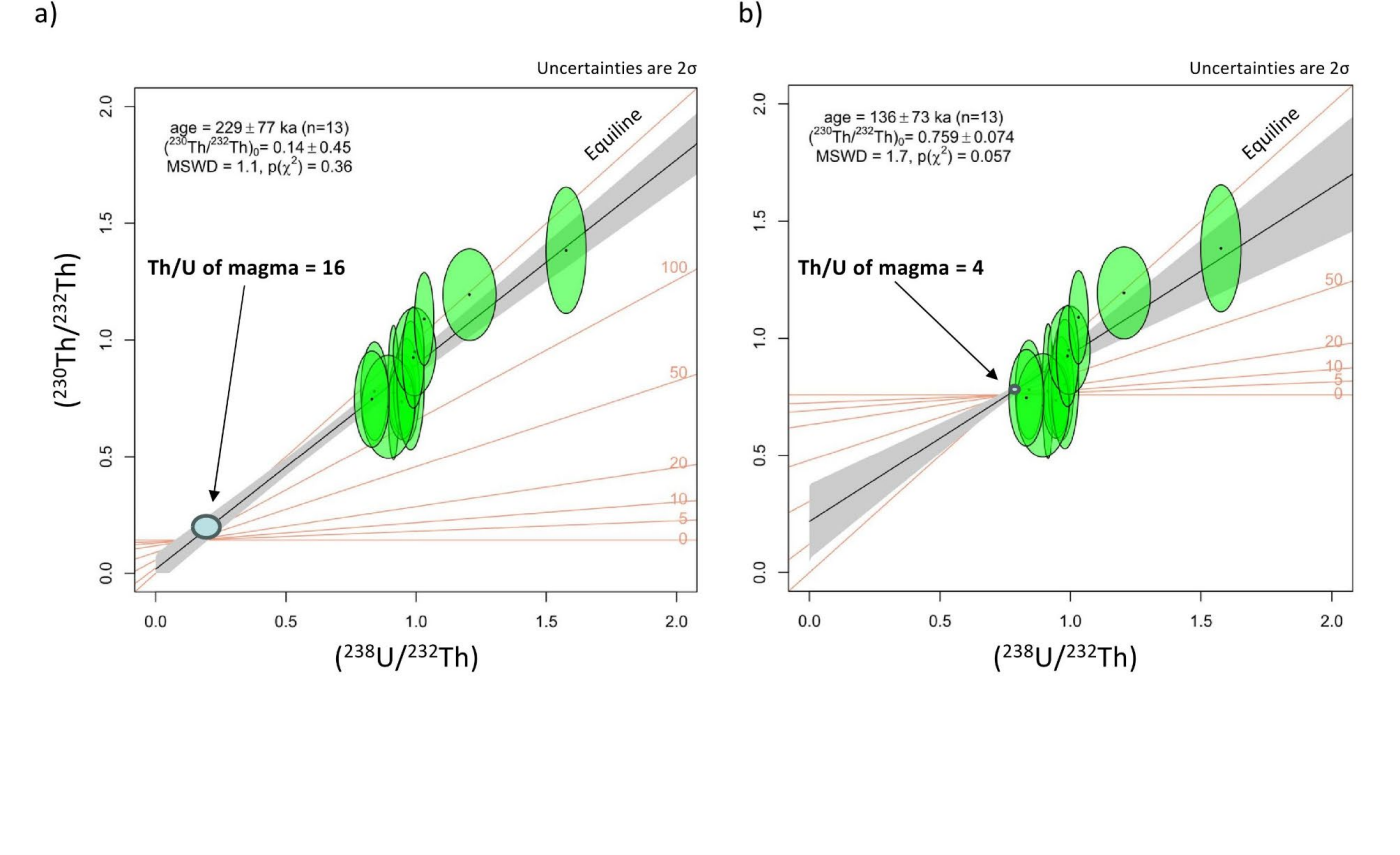
U-Th activity ratio diagram for zircons from the Akazaki lava, assuming Th/U of magma at 16.0 ± 1.6 (**a**) and at 4.00 ± 0.04 (**b**). IsoplotR^20^ was used to plot and calculate the age.

Ten out of 13 zircons from the Akazaki lava passed the U-Th criteria and yielded a weighted mean model age of 162 ± 30 ka and a median model age of 172 + 89/−24 ka (Table S2), assuming Th/U_magma_ of 16. In a U-Th activity ratio plot, all 13 zircons are above the 100 ka isochron and most of zircons are below the equiline (Fig. 4a). Assuming Th/U_magma_ of 16 with 10% uncertainty, an isochron age of 229 ± 77 ka (MSWD = 1.1) was obtained (Fig. 4a). This isochron age changes slightly to 229 ± 146 ka and 227 ± 59 ka, assuming Th/U_magma_ of 8 and 24 with 10% uncertainty, respectively. On the contrary, assuming Th/U_magma_ of 4 with 1% uncertainty, a U-Th isochron age of 136 ± 73 ka (MSWD = 1.7) was obtained (Fig. 4b). This age is significantly younger than the U-Pb and Th-Pb ages of ~ 250 ka and also some grains are plotted below the 50 ka isochron, which contradicts that the Akazaki lava is older than the 95 ka K-Tz. This corroborates that Th/U_magma_ of the Akazaki lava is significantly larger than 4. Note that assuming Th/U_magma_of 4 with 10% uncertainty, the IsoplotR^20^ could not calculate an isochron age.

### Trace element analysis

Zircon trace element analytical results are shown in Supplementary Table S3. Zircons with a high (> 5000 cps) P were omitted due to possible apatite inclusion and those with a high (> 10 ppm) K were deleted because of the high probability that they contained feldspar or melt inclusions. Chondrite-normalized rare earth elements (REEs) patterns for the three reference zircons are in good agreement with each reference value (Fig. S2), corroborating the validity of the analytical procedure.

**Fig. 5:**
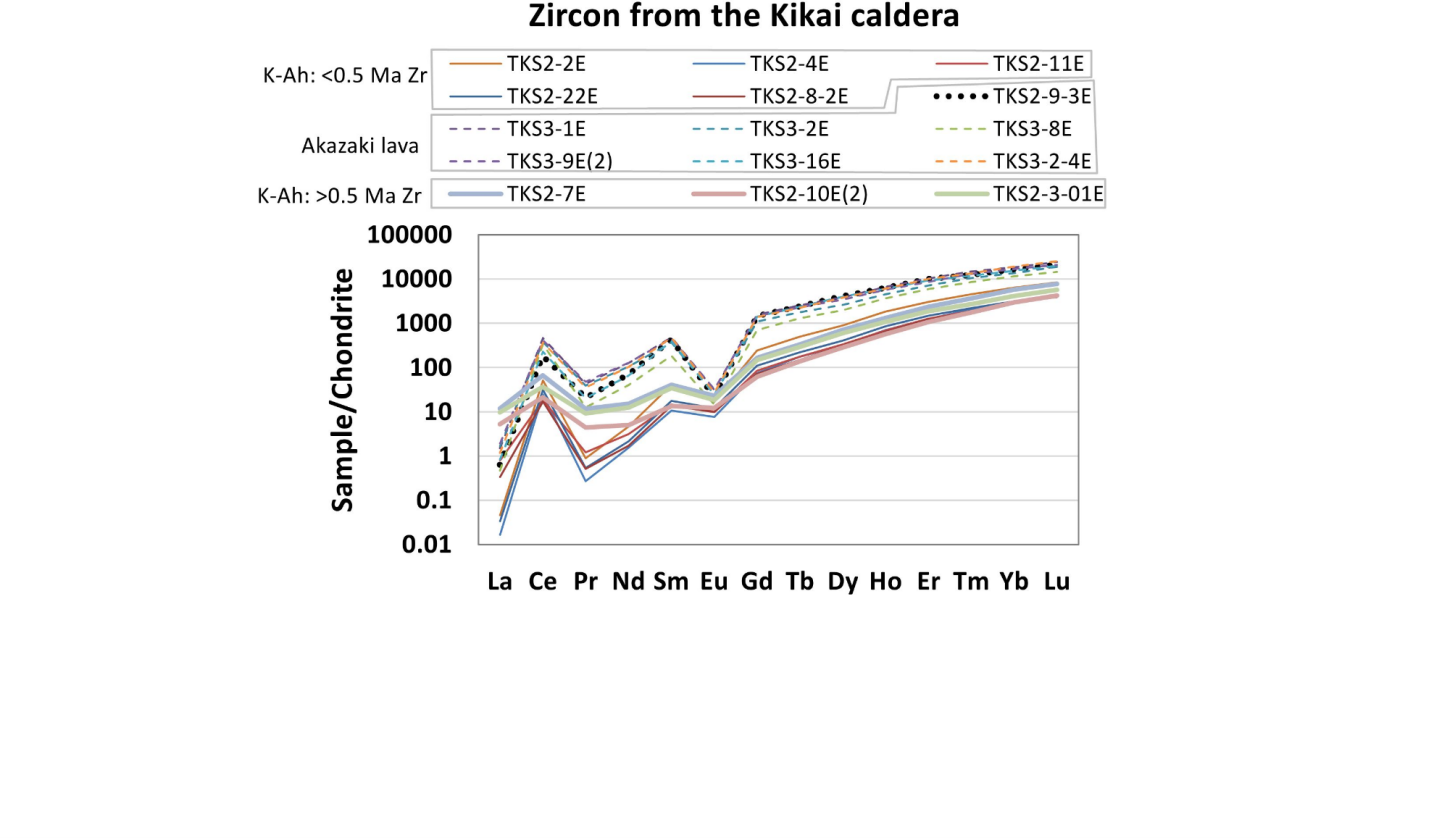
Chondrite-normalized REE patterns for zircon from the Kikai caldera (site: Takeshima Island). Zircons from K-Ah (TKS2) are shown in solid line except for KTZ2-9-3E. Zircons from the Akazaki lava (TKS3) are shown in dashed line. A dotted line (TKS2-9-3E) was from K-Ah but probably a xenocryst from the Akazaki lava. Zr: zircon.

**Fig. 6:**
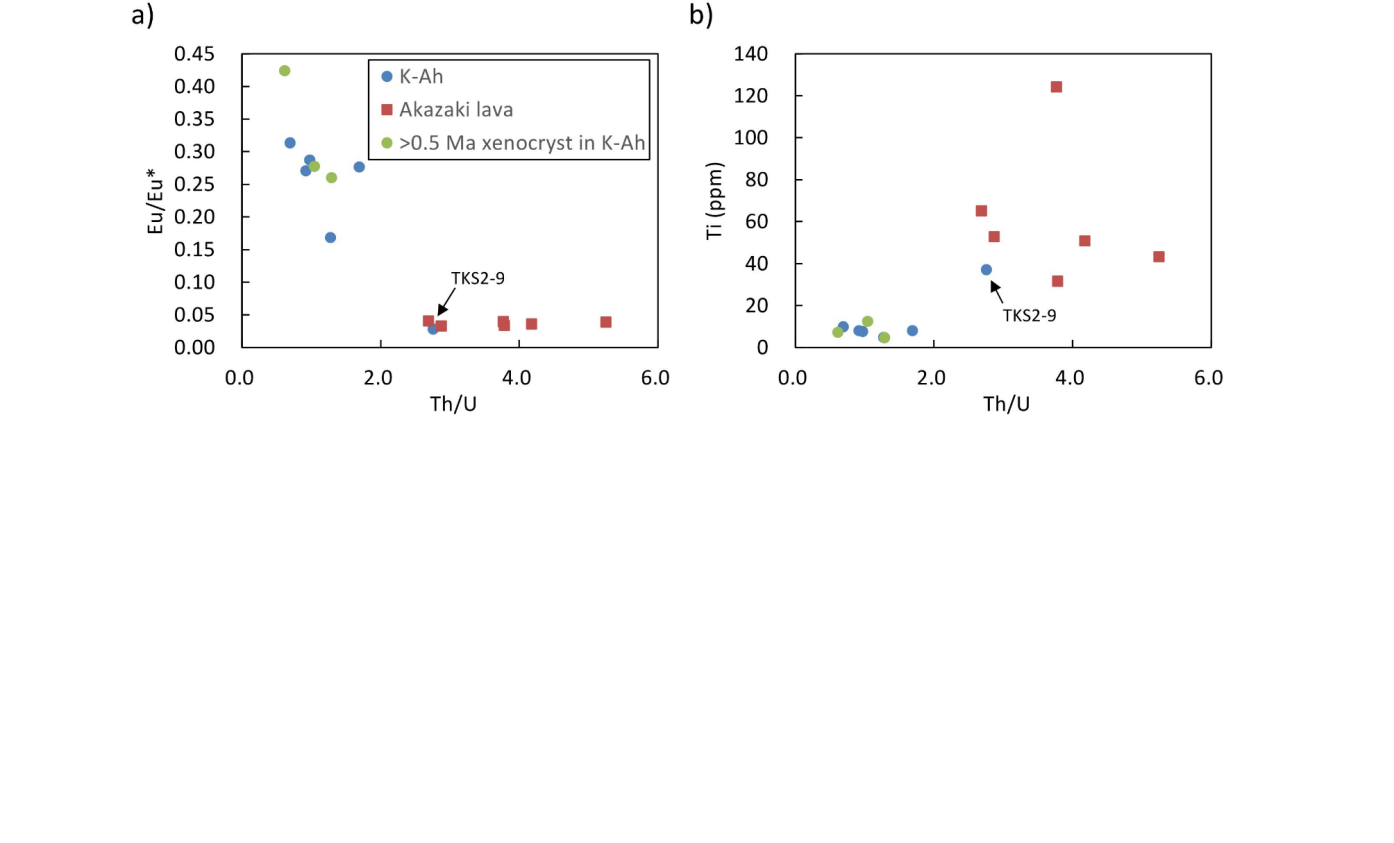
Covariations between (**a**) Eu/Eu* and (**b**) Ti with Th/U in zircon. Eu/Eu* were calculated using the equation (Eu/Eu* = [Eu_ch_]/([Sm_ch_]^0.5^*[Gd_ch_]^0.5^), where [X_ch_] are chondrite-normalized values in ppm.

Chondrite-normalized REE patterns for zircon from the Kikai caldera (Fig. 5) showed a distinctive feature between K-Ah and the Akazaki lava, in that zircons from the Akazaki lava have much more abundant REEs and a stronger negative Eu anomaly than those from the K-Ah. Note that a zircon from K-Ah (sample: TKS2-9) was in the range of the Akazaki lava (Figs. 5 and 6) therefore it is likely this grain is a xenocryst which originated from the Akazaki lava.

## Discussion

### Validity and usefulness of zircon Th-Pb dating using LA-ICP-MS

First of all, in this study we applied zircon Th-Pb dating using laser ablation-inductively coupled plasma-mass spectrometry (LA-ICP-MS), which has been seldom applied to Quaternary geochronology. Sakata et al.^21^ obtained a Th-Pb age of 0.767 ± 0.015 Ma (*n* = 31; MSWD = 1.5) for the Bishop Tuff zircon using LA-ICP-MS. They used age data that had no or negligible common Pb contamination. Therefore, they did not apply a common Pb correction. Here, we did a common Pb correction and obtained a Th-Pb age of 0.78 ± 0.08 Ma (*n* = 8; MSWD = 5.0) for the Bishop Tuff (Fig. S1), which is in agreement with that of Sakata et al.^21^ and the reference age of 0.767 ± 0.001 Ma^18^. Moreover, our Th-Pb age of 28.40 ± 1.40 Ma for the Fish Canyon Tuff zircon is in agreement with the its reference age of 28.402 ± 0.023 Ma^19^. However, our Th-Pb age of 384.0 ± 19.0 Ma for the Plešovice zircon is significantly older than its reference age of 337.13 ± 0.37 Ma^17^. Li et al.^22^ pointed out that ^208^Pb tends to be lost more readily than ^206^Pb in altered zircon, i.e., the Th-Pb system tends to be disturbed more easily than the U-Pb system. Here, we used common ^206^Pb value in lieu of common ^208^Pb for common Pb correction. We suppose that in old zircons such as Plešovice, the actual common ^208^Pb content is higher than its common ^206^Pb. For example, a Plešovice zircon portion (sample P3-2–384 in Table S1, which is the 384th laser pit data in a few large zircons) had a 375.8 ± 9.2 Ma using common ^206^Pb (*f*_206_%) of 1.5%, whereas it yields an age of 343.6 ± 9.2 Ma, assuming common ^208^Pb of 10.0%. Despite this limitation, we assume that our Th-Pb dating procedure is valid for young, especially Quaternary zircons, which broadens Th-Pb dating applicability to common Pb affected zircons.

As for the zircon Th-Pb ages for the K-Ah (Fig. 2), most individual Th-Pb ages have much larger uncertainty than their corresponding U-Pb ages, except for two grains (samples: TKS2-9 and TKS2-3–05) which have > 1,000 ppm Th (Table S1). The Th-Pb ages for the two grains are consistent with their U-Pb ages within uncertainty (Fig. 2), corroborating the validity and usefulness of the Th-Pb dating.

Zircons from the Akazaki lava yielded a weighted mean Th-Pb age of 0.24 ± 0.03 Ma, which is comparable with its U-Pb weighted mean age of 0.26 ± 0.03 Ma (Fig. 3). The Akazaki lava zircon has a high Th content of > 2,000 ppm and a high Th/U of > 2 (Table S1), therefore it demonstrates that in such a case Th-Pb method can yield comparable dates with the U-Pb method^22^. A zircon (sample: TKS3-10) yielded a U-Pb age of 0.80 ± 0.17 Ma, which we thought at first it was a xenocryst from the ~ 0.63 Ma Ksd or the ~ 0.73 Ma Anbo tephras, while its Th-Pb age was calculated to be 0.23 ± 0.19 Ma (Fig. 3). Therefore, this grain is likely an autocryst from the Akazaki lava magma. Although it is uncertain why this grain showed an anomalously old U-Pb age of ~ 0.8 Ma, it demonstrates the usefulness to calculate a Th-Pb age as well as U-Pb age to check internal consistency.

### Validity and usefulness of zircon U-Th dating using LA-ICP-MS

The only zircon (sample: TKS2-3–05; see a transmitted light image of this grain in Fig. 2 and a cathodoluminescence (CL) image in Fig. S3) from K-Ah that yielded a reliable U-Th age of 53 ± 26 ka (1σ) or 0.053 ± 0.052 Ma (2σ) agrees with its U-Pb age of 0.13 ± 0.07 Ma and Th-Pb age of 0.16 ± 0.05 Ma. Although these dates had a large uncertainty, the consistent ages validate that simultaneous triple (U-Pb, Th-Pb, U-Th) dating is useful to check internal consistency.

As for the Akazaki lava zircon, the weighted mean U-Th model age of 162 ± 30 ka (Table S2) is significantly younger than its corresponding U-Pb (262 ± 28 ka) and Th-Pb (242 ± 26 ka) ages. Although uncertainties are large, its median model age of 172 + 89/−24 ka (Table S2) and U-Th isochron age of 229 ± 77 ka (Fig. 4a) agree with the U-Pb and Th-Pb age of ~ 250 ka. Therefore, as Ito^23^ mentioned, simultaneous U-Pb and U-Th dating is useful to check internal consistency for young (< 0.4 Ma) zircons. In this study, simultaneous triple (U-Pb, Th-Pb, U-Th) dating is more useful than the double (U-Pb, U-Th) dating approach.

### Implications of the K-Ah zircon ages

Most zircon Th-Pb ages from K-Ah show a larger uncertainty than the corresponding U-Pb ages, therefore we do not discuss Th-Pb age for K-Ah in detail. Note that a zircon from K-Ah (sample: TKS2-9) was assumed to be derived from the Akazaki lava as shown in the REE pattern (Fig. 5). Its U-Pb age of 0.19 ± 0.05 Ma is recalculated to be 0.25 ± 0.03 Ma assuming Th/U_magma_ = 16.0 ± 8.0 (i.e., Akazaki lava magma), which is in good agreement with its Th-Pb age of 0.26 ± 0.04 Ma.

**Fig. 7:**
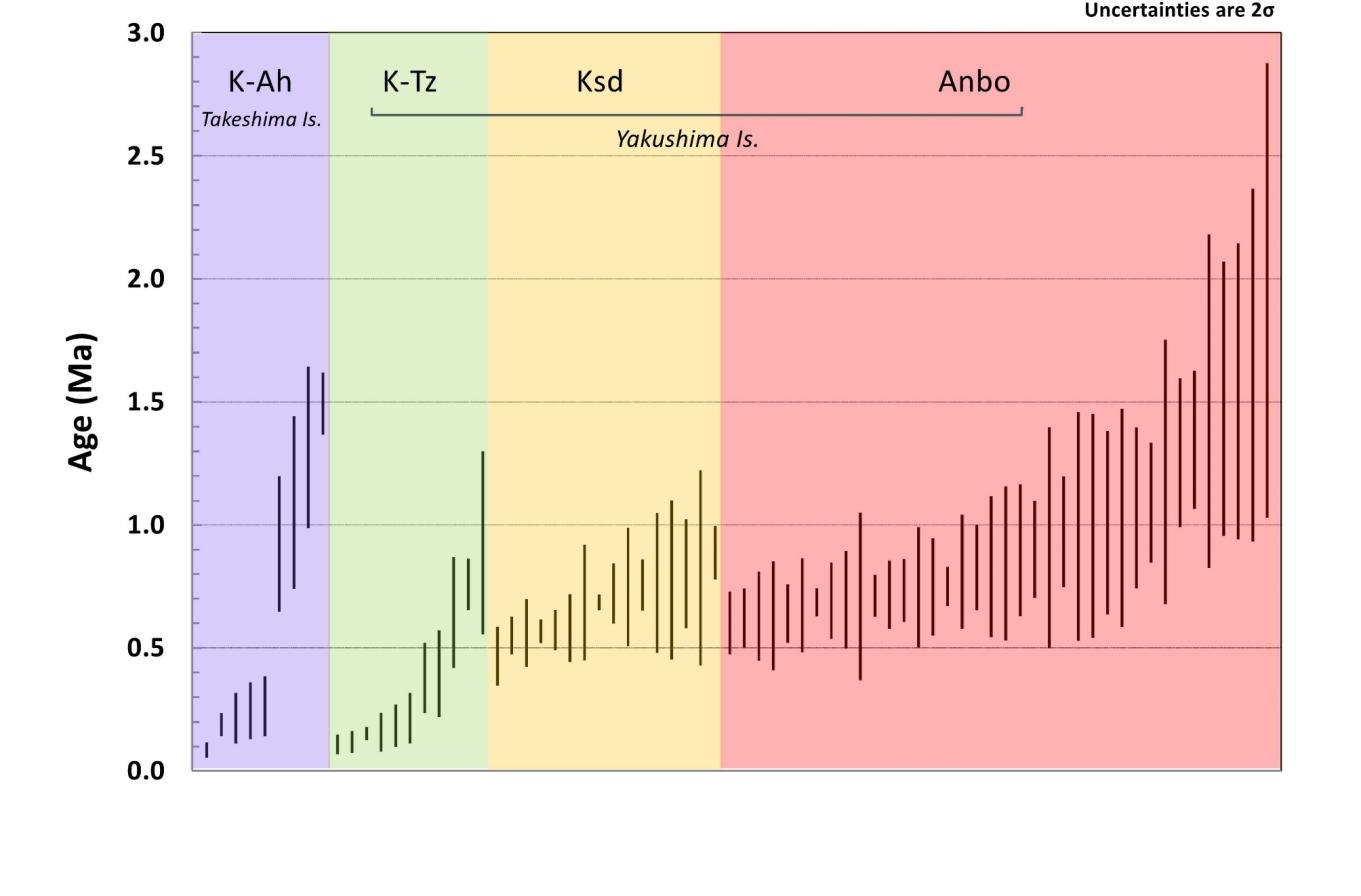
Zircon individual U-Pb (^238^U-^206^Pb) ages arranged in rank order for K-Ah, K-Tz, Ksd, and Anbo tephras. Ages with < 50% uncertainty (2σ) were plotted. K-Ah: this study, K-Tz, Ksd, and Anbo: Ito et al.^15^. Data are shown in Table S7.

Zircon U-Pb ages from the K-Ah tephra ranged from 0.09 ± 0.03 Ma to 1.49 ± 0.13 Ma (Fig. 2; Table S1). The youngest two grain ages (0.09 ± 0.03 Ma; 0.13 ± 0.07 Ma) are older than the 7.3 ka (or 0.0073 Ma) K-Ah and coeval with the 95 ka (or 0.095 Ma) K-Tz eruption. Dating younger than 0.1 Ma zircon by U-Pb is challenging and hence it is difficult to argue that they are autocrysts of the K-Ah magma or they are zircons associated with the K-Tz or ~ 0.14 Ma K-ab magmatism. With this limitation, it is still possible to argue that zircon-crystallizing magmatism was occurring at ~ 0.1 Ma in the Kikai caldera. More intriguing information can be derived from older zircons. To focus on U-Pb ages with better uncertainty, Fig. 7 shows ages with < 50% uncertainty for K-Ah from Takeshima (this study) and K-Tz, Ksd, and Anbo from Yakushima^15^. From K-Ah zircon U-Pb ages, it is evident that zircon-forming magmatism commenced at 1.5–1.0 Ma. Although we could not find 0.7–0.6 Ma zircons from K-Ah, K-Tz contained them, therefore Ito et al.^15^ assumed both Ksd and Anbo are originated from the Kikai caldera. The Anbo tephra also contains substantial 1.5–1.0 Ma zircons, which corroborates the assumption of 1.5–1.0 Ma magmatism in the Kikai caldera. Moreover, it is evident from the K-Ah and K-Tz U-Pb ages that zircon-crystallizing magmatism persisted from ~ 0.4 Ma to ~ 0.1 Ma.

### Implications of the Akazaki lava zircon ages

**Fig. 8:**
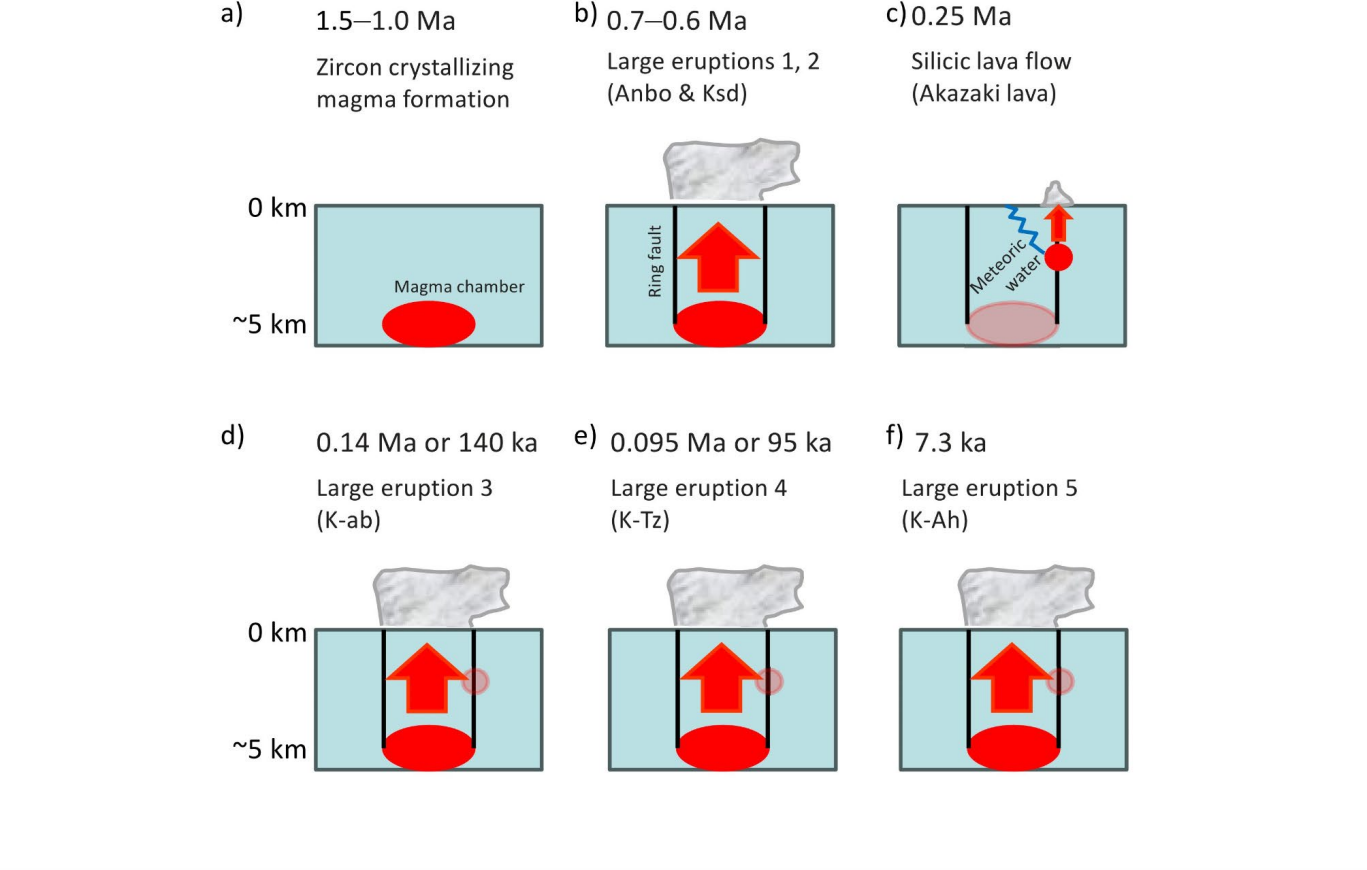
Schematics of the evolution of the Kikai caldera. (**a**) 1.5–1.0 Ma, (**b**) 0.7–0.6 Ma, (**c**) 0.25 Ma, (**d**) 0.14 Ma or 140 ka, (**e**) 0.095 Ma or 95 ka, (**f**) 7.3 ka. Red arrows indicate magma ascent. A blue zigzag line in (**c**) indicates the magma was affected by meteoric water infiltration.

Zircons from the Akazaki lava yielded weighted mean ages of 0.26 ± 0.03 Ma and 0.24 ± 0.03 Ma for U-Pb and Th-Pb (Fig. 3), respectively, yielding a combined weighted mean age of 0.25 ± 0.02 Ma (MSWD = 0.89). Although it indicates a zircon crystallization age, we assume that the Akazaki lava erupted at ~ 0.25 Ma (Table 1) because the magma was formed at a shallow crustal level as discussed below (Fig. 8c). As far as we know, this is the first case that a precise age older than the ~ 140 ka (or 0.14 Ma) K-ab eruption was determined for the direct sample from the Kikai caldera. It also demonstrates that a silicic magmatism occurred at ~ 250 ka (or 0.25 Ma) in the Kikai caldera (Fig. 8c).

### Implications of zircon trace elements

It was found that the Akazaki lava magma is unique in that zircons from the lava have much more abundant REEs and a stronger negative Eu anomaly than zircons from the K-Ah (Figs. 5 and 6a). The abundant REEs indicate the Akazaki lava magma was highly fractionated and the strong negative Eu anomaly is assumed that feldspar had been removed from the magma before zircon crystallization, both of which indicate that the Akazaki lava magma was a residue of crystallizing magma^24^ and hence it seems to have resided at relatively shallow crustal levels (Fig. 8c).

Although the sampled Akazaki lava was strongly altered (silicified) and it was impossible to obtain a reliable whole rock chemistry, the Akazaki lava Th/U_magma_ of 16 ± 8 was reasonably assumed from the high Th/U_zircon_ of ~ 3.7 (Tables S1, S3; see also Fig. 6) and the fact that the Th/U fractionation between zircon and silicic magma (i.e., *ƒ*_Th/U_ = (Th/U_zircon_)/(Th/U_magma_)) is commonly 0.2–0.3 (e.g., Sakata et al.^21^). High Th/U ratios of Akazaki lava magma may have been due to hydrothermal (in this case, silicic) alteration in the source region, because oxidizing meteoric water has a strong potential to mobilize U relative to Th during hydrothermal alteration^25^. This also indicates that the Akazaki lava magma resided at relatively shallow crustal levels where meteoric water may have affected the chemistry of the magma chamber prior to eruption (Fig. 8c).

Titanium (Ti) content of the Akazaki lava zircon is also remarkably higher than that of K-Ah (Fig. 6b), which indicates that the Akazaki lava magma was hotter than the magmas that crystallized zircons found in the K-Ah. Assuming the activities of SiO_2_ and TiO_2_ in the magma as 1 and 0.5 respectively, Ti-in-zircon thermometry^26^ calculates magmatic temperature at 741–839 ℃ for zircons found in the K-Ah (except for a xenocryst supposedly derived from the Akazaki lava) and at 954–1175 ℃ or 954–1062 ℃ for zircons from the Akazaki lava (Table S3). Note that the highest temperature of 1175 ℃ (sample TKS3-9) should be treated with caution because this is due to an anomalously high Ti content of 124 ppm (Table S3). This grain also shows an elevated Fe content of 407 ppm, which may indicate inclusions and contaminants in LA-ICP-MS analyses^27^. From the estimated magmatic temperatures and the zircon REE patterns (Fig. 5), it can be inferred that zircon-crystallizing magmas in the Kikai caldera were formed at a similar condition except for the Akazaki lava magma. Assuming that the first caldera-forming eruption occurred at 0.7–0.6 Ma, the Akazaki lava magma chamber may have been formed at shallow crustal levels via caldera ring faults (Fig. 8c). Besides the rhyolitic Akazaki lava, the basement of the Takeshima Island contains undated basaltic to dacitic lavas^14^. These lavas also may have effused from similar ring faults.

### Magmatic evolution and eruption history of the Kikai caldera

As discussed above, zircon crystallizing magmatism commenced at 1.5–1.0 Ma (Fig. 8a). Zircon chemistry indicates this magmatism is similar with other caldera-forming magmatism in the Kikai caldera (Figs. 5 and 6), therefore we assume that the magma resided at similar crustal levels with other caldera-forming magmatism of ~ 5 km in depth, because the depth of the K-Ah magma chamber is estimated at 3–7 km from the gas-saturation pressure of melt inclusions in plagioclase^28^. Note that the assumed magma chamber depth of ~ 5 km may have been a few km deeper because most subvolcanic magma chambers reside at 8 ± 2 km (or 2 ± 0.5 kbar)^29^.

Ito et al.^15^ assumed that the first large eruption occurred at 0.7–0.6 Ma at the Kikai caldera because the 95 ka K-Tz tephra from Yakushima Island (Fig. 1) contained 0.7–0.6 Ma zircons that seemed to be associated with caldera-forming widespread tephras (Ksd and/or Anbo). Although we could not have precise 0.7–0.6 Ma zircons from the K-Ah tephra, the evidence of 1.5–1.0 Ma magmatism seems to corroborate the assumption that the first large eruption happened at 0.7–0.6 Ma (Fig. 8b). It is likely that during the ~ 0.3–0.9 m.y. time between initiation of magmatism dated by zircons crystal ages, and the first caldera forming eruption, there were eruptions and magma differentiation in the crust promoting enrichment of magma in volatiles toward a large eruption.

At 0.25 Ma the silicic Akazaki lava eruption occurred at shallower levels than the caldera-forming magma chamber as discussed (Fig. 8c). This is similar with post 7.3 ka K-Ah magmatism, because post K-Ah rhyolites and basalts were produced along the caldera rim at shallower levels than the K-Ah magma chamber^28^. After 0.25 Ma, three caldera-forming eruptions at 140 ka, 95 ka, and 7.3 ka ensued at the Kikai caldera (Fig. 8d–f; Table 1).

## Methods

### Sample description

A pyroclastic flow deposit, the Takeshima Pyroclastic Flow Deposit^14^ or Takeshima ignimbrite^8^, was sampled (sample name: TKS-2) (Fig. 1b and c). It is a non-welded deposit of well-vesiculated white rhyolitic pumice and matrix of white vitric ash (Fig. 1c), mainly found in Takeshima Island with a maximum thickness of ~ 30 m^8,14^. The K-Ah tephra is a facies of air-fall fine ash from the ash cloud of the Takeshima ignimbrite. The K-Ah contains plagioclase, clinopyroxene, orthopyroxene, magnetite, and ilmenite as phenocrysts^14^, which was also confirmed by SEM-EDS analyses in this study. The SiO_2_ content of glass shards from TKS-2 was ~ 73% (*n* = 7), while that from K-Tz in Yakushima Island was ~ 78% (*n*= 6) (Table S4). These data are in accord with those reported in Machida and Arai^12^, confirming that the sampled TKS-2 is from K-Ah.

A highly silicified rhyolitic lava (Fig. 1e), the Akazaki lava, > 60 m thick at a cliff of Komoriko Port (Fig. 1b and d), was sampled (sample name: TKS-3). The sample contains a very small amount of phenocryst (< 1% in total), including quartz, plagioclase, clinopyroxene, orthopyroxene, and magnetite, which was confirmed by SEM-EDS analyses.

### Sample preparation

Zircons were separated from the two samples (each ~ 1 kg) using standard heavy liquid and magnetic techniques. To purify zircons, separated minerals were immersed in HF (~ 46%) and then in HCl (~ 10%) both for a few days at room temperature. A small amount (30–40 grains) of zircons were obtained from each sample. Most zircons are euhedral and generally smaller than 200 μm in size (Figs. 2 and 3). Zircon grains were handpicked and embedded in a PFA Teflon sheet (0.5 mm thick). Two Teflon sheets (sample name: TKS2, TKS2-3) for the K-Ah and two sheets (TKS3, TKS3-2) for the Akazaki lava were prepared. No polishing was performed for the initial dating procedure. After the dating, zircons were polished to a 1 μm finish using diamond paste and CL images were obtained using a Hitachi TM4000Plus electron microscope (Fig. S3).

### U-Pb dating

LA-ICP-MS analyses were performed at the Central Research Institute of Electric Power Industry (CRIEPI), Japan, using a Thermo Fisher Scientific ELEMENT XR magnetic sector-field ICP-MS (SF-ICP-MS) coupled to a New Wave Research UP-213 Nd-YAG laser. Data were acquired at four occasions in 2017, 2023 and 2024 using instrumental parameters as shown in Table S5. Key features are as follows: In order to acquire U-Pb, Th-Pb and U-Th ages simultaneously, the normal dating procedure for U-Pb was changed in that ^230^Th was measured instead of ^235^U. N_2_ gas was added to increase signal intensity for 2023 and 2024 experiments. The instrument was tuned daily to obtain ^232^Th signal intensity of > 500,000 cps and ThO/Th of < 0.8% using NIST SRM 613 with a 30 μm beam diameter, 10 Hz repetition, and ~ 9 J/cm^2^ laser fluence at a 5 μm/s scanning speed for the 2023 and 2024 experiments. Samples were ablated in helium gas by pulses at a 10 Hz repetition rate with 7–8 J/cm^2^ laser fluence and a 30 μm beam diameter. The focus of the laser beam was fixed at the sample surface throughout the data acquisition. Data were acquired in electrostatic scanning (E-scan) mode over 1080 mass scans over a 30 s background measurement, followed by 30 s sample ablation and then a 45 s background measurement.

Data for the first 10 s of ablation were neglected to avoid surface Pb contamination and signal instability, and the 10–20 s laser ablation data and the 20–30 s data were adopted as shallow and deep sections data, respectively. Approximately 24 μm in depth were drilled during the 30 s laser ablation and therefore the shallow section age is derived from material ablated between ~ 8 and 16 μm depths and ~ 16 to 24 μm depths for the deep section.

Individual U-Pb ages were corrected for common-Pb using a modified ^207^Pb-based method^30^ adopting the common ^207^Pb/^206^Pb of 0.8357 from the Pb isotope evolution model^31^ and measured ^207^Pb/^206^Pb. The modified ^207^Pb method employs both Th/U and Pa/U partitioning (*f*_Th/U_ and *f*_Pa/U_, respectively) for correcting initial ^230^Th and ^231^Pa disequilibrium in the mineral-magma system. The *f*_Th/U_ was calculated assuming Th/U_magma_ was 4.0 ± 2.0 and employing individually measured Th/U of zircon for the K-Ah. The reason to choose Th/U_magma_of 4.0 ± 2.0 is because it covers most of silicic magma Th/U ratio^32^, yielding *f*_Th/U_ of mostly < 0.5 (Table S1). As for the Akazaki lava magma, Th/U_magma_ of 16.0 ± 8.0 was adopted, yielding *f*_Th/U_ of mostly ~ 0.2 (Table S1). The measured *f*_Th/U_ with a 50% uncertainty and an assumed *f*_Pa/U_ of 3.36 ± 0.40 (ref: 30) were used for all age calculations. Data with a high common Pb contamination (*f*_206_%) of > 75% were excluded for further analyses^23^ and the MSWD^33^ is used as a statistical test of validity of weighted mean ages. The 91500 zircon (1062 ± 15 Ma^34^) was used as a primary reference material with zircons of Plešovice (337.13 ± 0.37 Ma^17^), Bishop Tuff (0.767 ± 0.001 Ma^18^) and Fish Canyon Tuff (28.402 ± 0.023 Ma^19^) used as secondary reference materials. Th/U_magma_of 3.0 ± 1.5 for the Bishop Tuff^18^ and that of 2.2 ± 1.1 for the Fish Canyon Tuff^19^ were adopted, yielding *f*_Th/U_ of ~ 0.2–0.3 (Table S1). Uranium and Th concentrations were quantified by comparing counts of ^238^U and ^232^Th for the sample relative to the 91500 zircon reference material, which is assumed to have homogeneous U and Th concentrations of 80 and 30 ppm respectively^34^, followed by a drift correction relative to the NIST SRM 610 glass standard. No down-hole isotopic (Pb/U, Th/U) fractionation correction was performed because data from the same depth range (or time span) were used for standards and unknowns in each analysis^23^.

In order to check reproducibility of and augment the K-Ah data, some selected zircons were dated twice using polished surface and a laser diameter of 25 μm (Table S1; Fig. S3).

### Th-Pb dating

Th-Pb (^232^Th-^208^Pb) dating was performed using values of ^232^Th and ^208^Pb obtained during the U-Pb dating procedure, i.e., simultaneous Th-Pb and U-Pb dating was performed in this study. In the Th-Pb dating, because there are no long-lived intermediate daughter nuclides in the ^232^Th-^208^Pb decay system, it is possible to neglect initial disequilibrium correction^21,35^. Here, correction of common Pb was performed assuming that the same common Pb contamination occurred both for U-Pb (^238^U-^206^Pb) and Th-Pb (^232^Th-^208^Pb) systems, and employed the *f*_206_% (Table S1) value for common Pb correction. The other procedures followed the U-Pb dating approach.

### U-Th dating

U-Th ages were obtained following Ito^23^, which shows a simultaneous U-Pb and U-Th dating approach using LA-ICP-MS. Key features are as follows. Firstly, ^230^Th/^238^U activity ratios or (^230^Th/^238^U) were measured for all reference zircons expected to be in secular equilibrium (i.e., 91500, Plešovice, Bishop Tuff and Fish Canyon Tuff, which are all > 0.4 Ma). The weighted mean of (^230^Th/^238^U) for all the reference zircons was calculated as a correction factor and then the (^230^Th/^238^U) for unknown sample was calculated using this correction factor^36,37^. U-Th dating was performed on data obtained in 2023 and 2024 experiments, because these data satisfied the experimental conditions of Ito^23^ especially with a condition of ThO/Th ratio of < 0.8% using NIST SRM 613.

Data with low ^230^Th counts (i.e., ^230^Th < 30 cps) were omitted to reduce the effect of molecular (zirconium oxides) interferences on ^230^Th. The abundance sensitivity on ^230^Th from much abundant ^232^Th was corrected using the “2.5 ppm correction” of Ito^23^.

Zircon isochron and model ages were obtained using IsoplotR v.5.5 of Vermeesch^20^. The model ages were calculated assuming Th/U of magma to be 4.0 for K-Ah and 16.0 for Akazaki lava. Data with a high (> 100% at 2σ) uncertainty were excluded for weighted mean age and median age calculations (Table S2). The weighted mean and median ages were calculated using Isoplot 4.15 of Ludwig^33^.

### Trace element analysis

Multiple element analysis of zircon was performed using the same LA-ICP-MS used for zircon dating, employing experimental conditions shown in Table S6. Key features are as follows. Thirty-five elements from ^23^Na to ^248^Cm were analyzed. Each analysis incorporated a background acquisition of 30 s (gas blank) followed by a 30 s data acquisition and a 45 s washout. Data were obtained at a 10–20 s (40–50 s from the start) time span. Corrections were made for mass bias drift which was evaluated by reference to the NIST 613 glass standard. Element concentrations were obtained by normalizing count rates for each analyzed element to those for SRM 613 (ref: 38) and Si, assuming SiO_2_ to be stoichiometric with a concentration of 32.7 wt% in zircon. Phosphorus (P), K, Ca, Ti, Fe and Th were monitored to evaluate possible involvements of mineral inclusions (apatite, K-feldspar, plagioclase, titanite, pyrite and monazite, respectively) in zircons or to monitor spikes. As a check of reproducibility, precision and accuracy, the NIST 610 glass standard was also analyzed. Moreover, 91500 zircon^38^, MAD-559 zircon^39^ and Bishop Tuff zircon^40^ were analyzed as secondary zircon standards in every 5–10 unknown measurements.

### SEM-EDS analysis

Typical minerals and glass left behind after zircon separation were mounted in epoxy and sectioned. Chemical composition was analyzed by SEM-EDS using Hitachi TM4000Plus electron microscope equipped with an Oxford AZtecOne EDS system, under an acceleration voltage of 15 kV and a working distance of ~ 10 mm. To monitor analytical accuracy and precision, we analyzed ATHO-G^38^ and glass shards of Aira-Tn (AT) tephra^12^ as working standards. The oxides composition results of glass shards are shown in Table S4.

## Electronic supplementary material

Below is the link to the electronic supplementary material.

Supplementary Material 1

Supplementary Material 2

## Data Availability

Data are available in an Excel file as Supplementary data.
