# Supplementary material for: Magmatic evolution of the Kikai caldera revealed by zircon triple dating and its chemistry
Source: Sci Rep. 2025 Jan 23;15:2968. doi: 10.1038/s41598-025-87264-5 (PMC11757711; doi:10.1038/s41598-025-87264-5)
Supplement: Supplementary file 1 — Supplementary Material 1 [file 41598_2025_87264_MOESM1_ESM.pdf]

Magmatic evolution of the Kikai caldera revealed by zircon triple dating and its chemistry

Hisatoshi Ito[1*]

[1]*Central Research Institute of Electric Power Industry, Chiba 270-1194, Japan*

Supplementary Figures 1–3

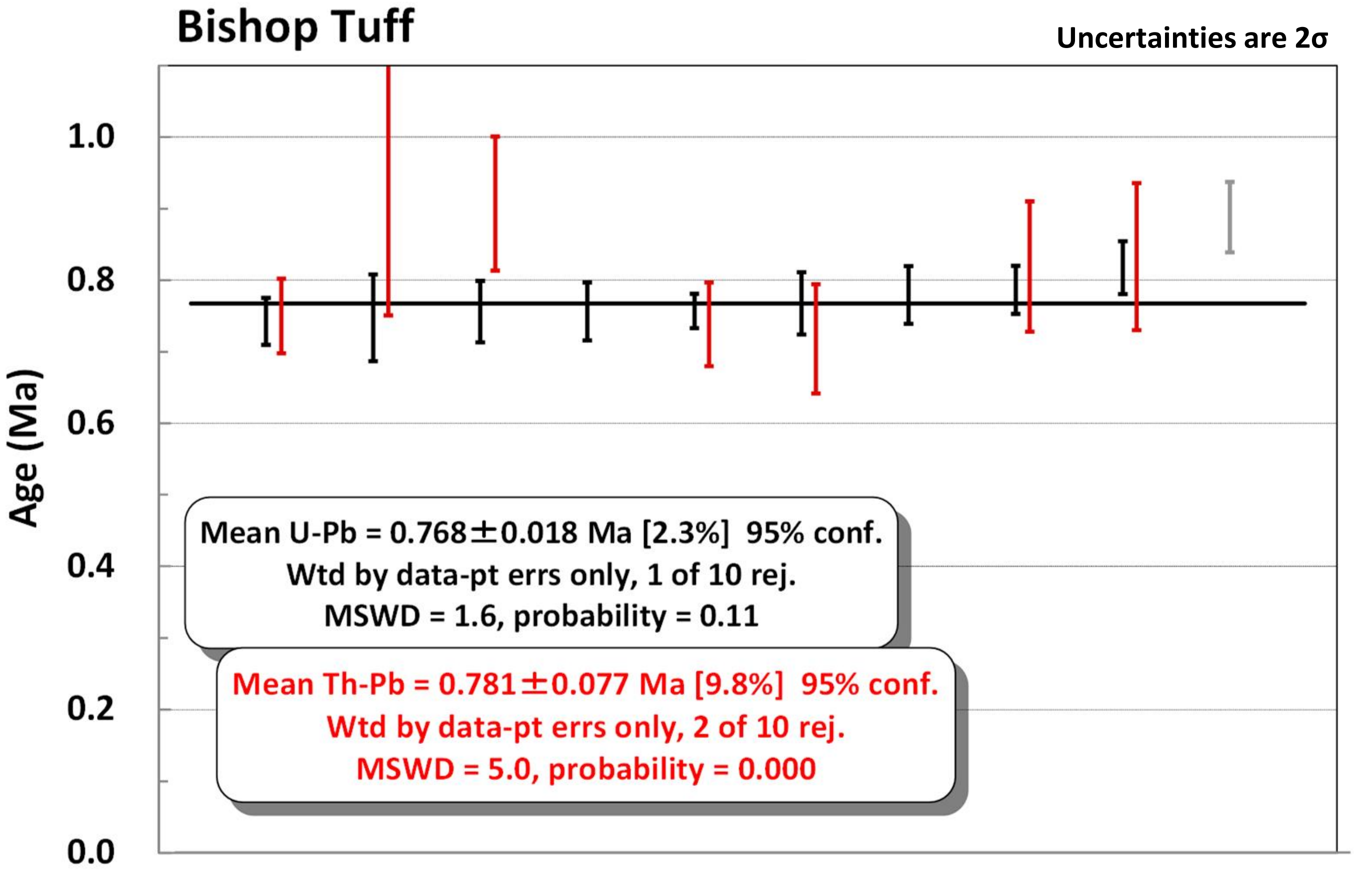

Fig. S1. Bishop Tuff zircon individual U-Pb (^{238}U-^{206}Pb) and Th-Pb (^{232}Th-^{208}Pb) ages arranged in rank order for U-Pb. Th-Pb ages are in red and arranged next to the corresponding U-Pb ages. A black horizontal bar shows the mean age of U-Pb. A gray vertical bar is omitted for the mean calculation.

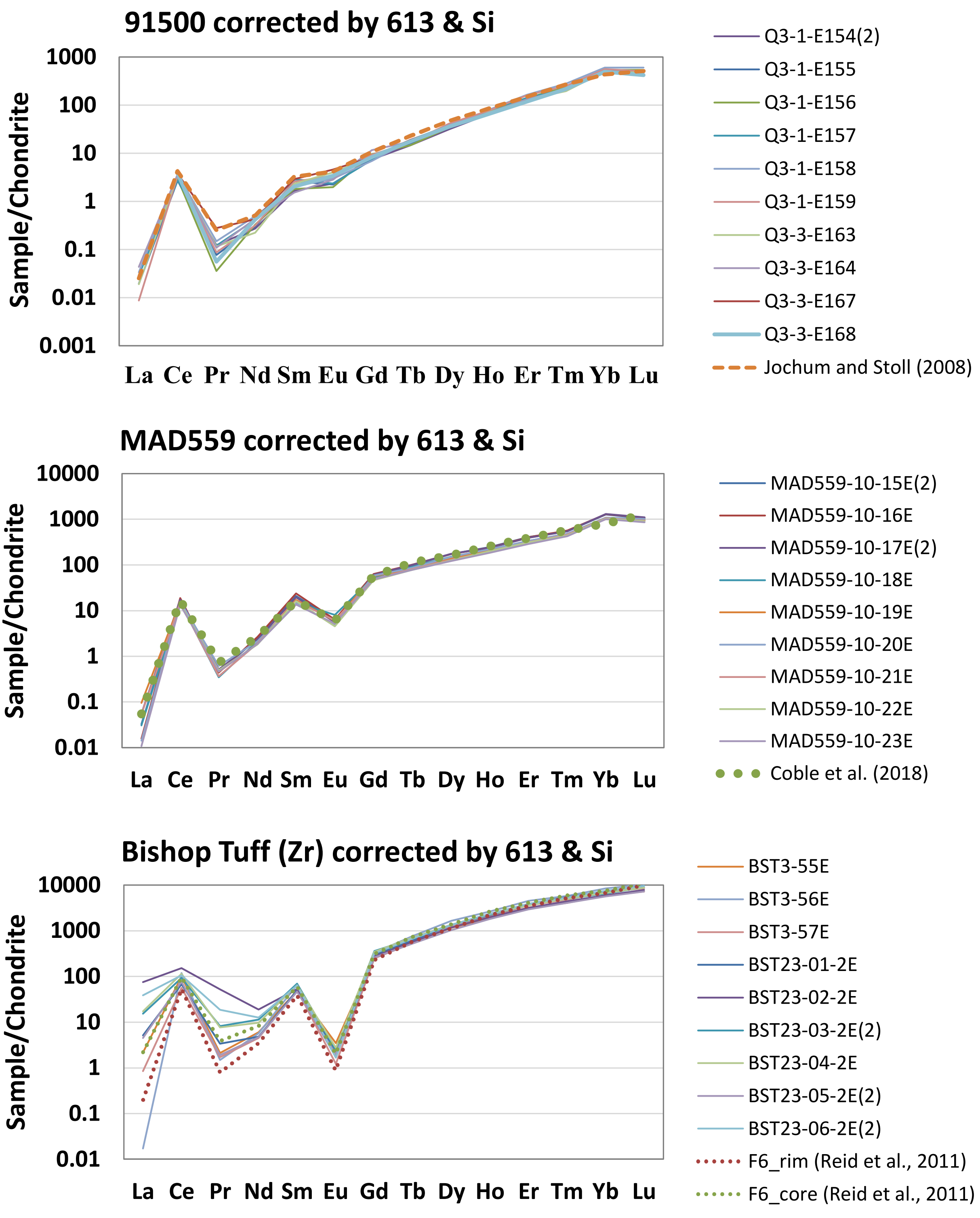

Fig. S2. Chondrite-normalized REE patterns for zircon from the three reference zircons (91500, MAD559, Bishop tuff).

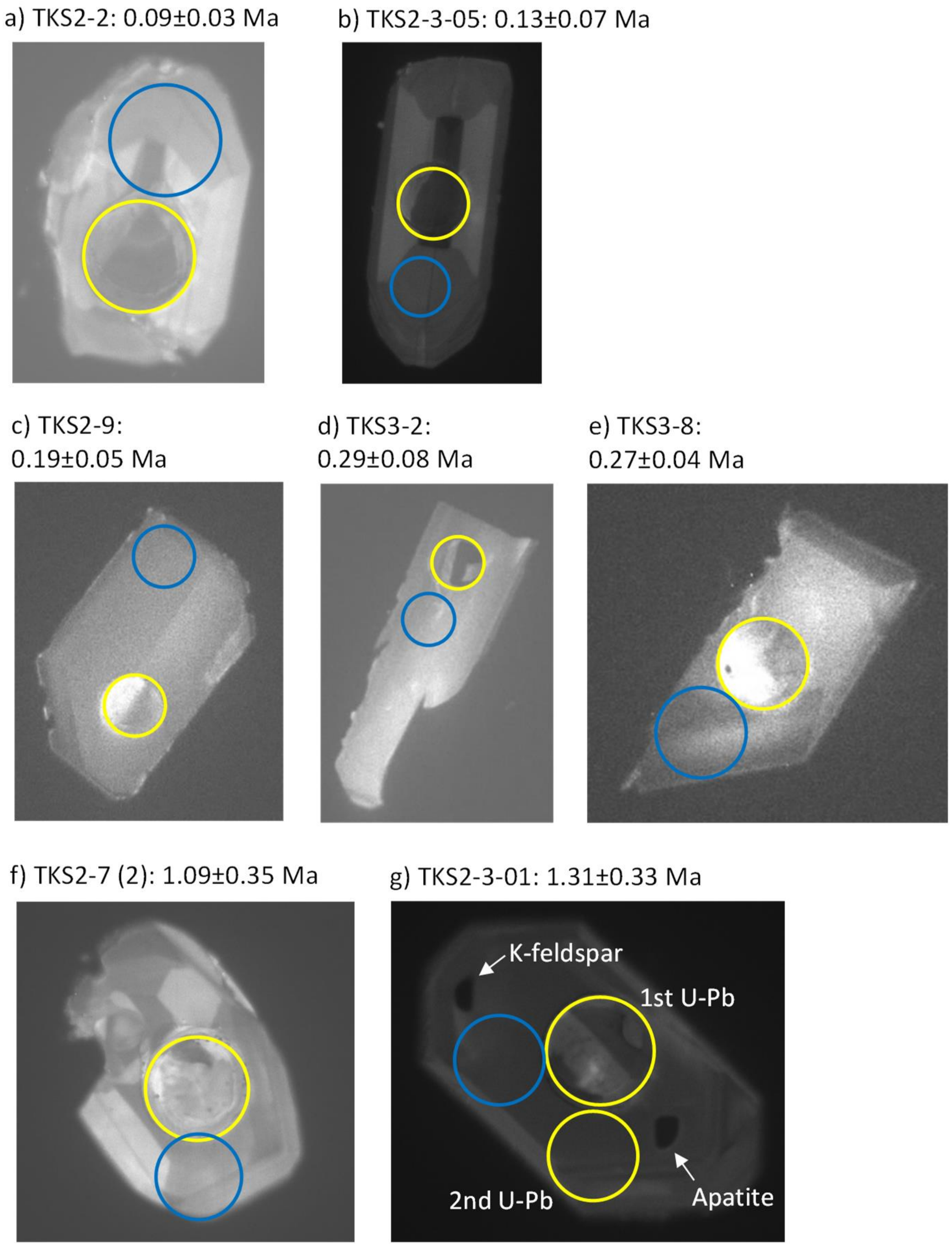

Fig. S3. Representative cathodoluminescence (CL) images of zircons from the Kikai caldera (site: Takeshima Island). a) and b) autocrysts? from the 7.3 ka K-Ah. c) xenocryst? from 0.25 Ma Akazaki lava contained in K-Ah, d) and e) autocrysts from Akazaki lava, f) and g) 1.5–1.0 Ma xenocrystic zircons contained in K-Ah. Zircon grain name and the U-Pb (^{238}U-^{206}Pb) age with 2σ uncertainty are indicated. Yellow and blue circles are where lasers were targeted for U-Pb and trace element analyses, respectively. Yellow circles (except smaller one in TKS2-3-01) are 30 μm in diameter, which also serve as a scale. Corresponding transmitted light microscopy images of these zircons excluding TKS2-7 are shown in Figs. 2 and 3. CL images were obtained using a Hitachi TM4000Plus electron microscope.
